# Kuoxin Decoction promotes lymphangiogenesis in zebrafish and *in vitro* based on network analysis

**DOI:** 10.3389/fphar.2022.915161

**Published:** 2022-08-11

**Authors:** Longping Peng, Mengjiao Ma, Yidan Dong, Qiong Wu, Shiying An, Min Cao, Yi Wang, Chang Zhou, Maolin Zhou, Xu Wang, Qianqian Liang, Youhua Wang

**Affiliations:** ^1^ Longhua Hospital, Shanghai University of Traditional Chinese Medicine, Shanghai, China; ^2^ Shanghai Cancer Center, Fudan University, Shanghai, China; ^3^ Spine Institute, Shanghai University of Traditional Chinese Medicine, Shanghai, China; ^4^ Key Laboratory of Theory and Therapy of Muscles and Bones, Ministry of Education, Shanghai University of Traditional Chinese Medicine, Shanghai, China

**Keywords:** Kuoxin Decoction, lymphangiogenesis, zebrafish, lymphatic endothelial cells, salvianolic acid B, network analysis

## Abstract

**Background:** Inadequate lymphangiogenesis is closely related to the occurrence of many kinds of diseases, and one of the important treatments is to promote lymphangiogenesis. Kuoxin Decoction (KXF) is an herbal formula from traditional Chinese medicine used to treat dilated cardiomyopathy (DCM), which is associated with lymphangiogenesis deficiency. In this study, we comprehensively verified whether KXF promotes lymphangiogenesis in zebrafish and *in vitro* based on network analysis.

**Methods:** We performed virtual screening of the active compounds of KXF and potential targets regarding DCM based on network analysis. Tg (Flila: EGFP; Gata1: DsRed) transgenic zebrafish embryos were treated with different concentrations of KXF for 48 h with or without the pretreatment of MAZ51 for 6 h, followed by morphological observation of the lymphatic vessels and an assessment of lymphopoiesis. RT-qPCR was employed to identify *VEGF-C*, *VEGF-A*, *PROX1*, and *LYVE-1* mRNA expression levels in different groups. After the treatment of lymphatic endothelial cells (LECs) with different concentrations of salvianolic acid B (SAB, the active ingredient of KXF), their proliferation, migration, and protein expression of VEGF-C and VEGFR-3 were compared by CCK-8 assay, wound healing assay, and western blot.

**Results:** A total of 106 active compounds were identified constituting KXF, and 58 target genes of KXF for DCM were identified. There were 132 pathways generated from KEGG enrichment, including 5 signaling pathways related to lymphangiogenesis. Zebrafish experiments confirmed that KXF promoted lymphangiogenesis and increased *VEGF-C* and *VEGF-A* mRNA expression levels in zebrafish with or without MAZ51-induced thoracic duct injury. In LECs, SAB promoted proliferation and migration, and it could upregulate the protein expression of VEGF-C and VEGFR-3 in LECs after injury.

**Conclusion:** The results of network analysis showed that KXF could regulate lymphangiogenesis through *VEGF-C* and *VEGF-A*, and experiments with zebrafish confirmed that KXF could promote lymphangiogenesis. Cell experiments confirmed that SAB could promote the proliferation and migration of LECs and upregulate the protein expression of VEGF-C and VEGFR-3. These results suggest that KXF promotes lymphangiogenesis by a mechanism related to the upregulation of VEGF-C/VEGFR-3, and the main component exerting this effect may be SAB.

## Introduction

Lymphangiogenesis is defined as the formation of new lymphatic vessels from existing lymphatic vessels, which involves the proliferation, migration, and tube formation of lymphatic endothelial cells (LECs). Although LECs are static in most cases, LECs migrate, proliferate, and form lymphatic vessels under the influence of growth chemokines when involved in inflammation, tumors, trauma, etc. Studies have confirmed that many diseases are associated with morphological or functional defects in the lymphatics such as lymphedema (Oliver et al., 2020), dilated cardiomyopathy (DCM) (Benvenuti et al., 2010), obesity (Blum et al., 2014; Escobedo et al., 2016), myocardial infarction (Angeli and Harvey, 2015; Henri et al., 2016), Parkinson’s disease (Zou et al., 2019), and stroke (Yanev et al., 2020).

In recent years, lymphatic development has drastically progressed with the identification of specific lymphatic endothelial cell (LEC) markers such as vascular endothelial growth factor receptor (VEGFR)-3, LYVE-1, PROX1, and Podoplanin. Many studies indicated that therapeutic lymphangiogenesis could be a new strategy for the treatment of diseases associated with defective lymphangiogenesis (Henri et al., 2016; Vuorio et al., 2017). A study showed that myocardial edema, inflammation, and fibrosis were reduced, cardiac function was improved, and the number of lymphatic vessels was increased in a model of myocardial infarction with treatment with VEGF-C (Houssari et al., 2020).

Dilated cardiomyopathy is one of the primary cardiovascular diseases and is one of the three leading causes of heart failure and sudden death. In recent years, many studies (Benvenuti et al., 2010) have confirmed that lymphatic capillaries are rare and the diameter of myocardial lymphatic vessels has decreased in myocardial interstitial fibrosis in patients with dilated cardiomyopathy. Therapeutic lymphangiogenesis may be a promising new approach for the treatment of DCM-induced heart failure (Peng et al., 2020), but there are few effective and safe drugs promoting lymphangiogenesis.

Kuoxin Decoction (KXF) is a traditional Chinese medicine (TCM) formula used for the treatment of DCM, the principal herbs of which are *Astragalus mongholicus* Bunge (Huangqi), *Polygonatum kingianum* Collett and Hemsl (Huangjing), *Salvia miltiorrhiza* Bunge (Danshen), *Neolitsea cassia* (L.) Kosterm (Guizhi), and *Trichosanthes kirilowii* Maxim (Gualoupi). A clinical trial (Wang et al., 2017) indicated that Western medicine combined with KXF could significantly improve the cardiac function and quality of life of patients with DCM compared with western medicine alone. Moreover, the combination therapy group showed a significantly lower readmission rate in some patients, indicating that the long-term efficacy of the combination therapy was better than that of the control group. However, the pharmacological mechanism of KXF and whether the therapeutic effect is related to lymphangiogenesis remain unclear. Therefore, this study aims to investigate whether the therapeutic effect of KXF is related to lymphangiogenesis.

Network analysis systematically explores the relationship between drugs and diseases based on an approach of “multi-gene and multi-target– complex disease.” It comprehensively reveals the mechanism of action of traditional Chinese medicine. Therefore, it has been considered a promising method for TCM research (Xu et al., 2011). As a spinal model animal, the zebrafish model is widely used in studies of cardiovascular diseases with features such as heart rate, cardiac action potential shape, duration, and diastolic heart function that are closer to the human heart than in rodents (Iorga et al., 2011; Wilkinson et al., 2014; Liu et al., 2016; Vornanen and Hassinen 2016; Zhao et al., 2018). Therefore, this study predicted the potential targets related to lymphangiogenesis of KXF for DCM based on the network analysis and verified the mechanism of regulating lymphangiogenesis in zebrafish models and lymphatic endothelial cells.

## Materials and methods

### Materials and reagents

KXF was provided by the TCM Preparation Department of Longhua Hospital, Shanghai University of Traditional Chinese Medicine. Salvianolic acid B (A0056) was purchased from CHENGDU MUST BIO-TECHNOLOGY (China).

Fetal bovine serum (FBS) (10100147), penicillin–streptomycin (15140122), and trypsin-EDTA (0.25%) phenol red (25200056) were collected from Gibco (America). MEM-ALPHA (01-042-1A) was obtained from Biological Industries (Israel). DMSO (D2650), chloroform (C2432), isopropanol (563935), PTU (P7629), tricaine (A5040), MAZ51 (676492), methylcellulose (M0512), tri reagent (T9424), and an anti-GAPDH (G9545) antibody were purchased from Sigma-Aldrich (America). DEPC-treated water (B501005) was purchased from Sangon Biotech (China). QuantiNova SYBR Green PCR Kit and QuantiNova Reverse Transcription Kit were provided by QIAGEN (Germany). Cell Counting Kit-8 reagent (C0038) and BeyoECL Plus (P0018S) were purchased from Beyotime (China). Anti-VEGFC antibody (ab83905), anti-FLT4/VEGFR3 antibody (ARG58698), and anti-rabbit IgG, HRP-linked antibody (7074S) were purchased from Abcam (England), Arigo (China), and Cell Signaling TECHNOLOGY (America), respectively.

### High-performance liquid chromatography

The extract of KXF (122.2 mg) was dissolved in 50% methanol (10 ml), the contents of salvianolic acid B (SAB) in which were determined, and the chromatographic separation conditions were as follows: Welch Ultimate XB-C18 column (4.6 × 250 mm, 5 μm); the mobile phase: AcCN (A)–0.3% phosphoric acid-H2O (B) (0–10 min, 5–20%A; 10–25 min, 20–40%A; 25–30 min, 40–95%A); flow rate: 1.0 ml/min. The detection wavelength of salvianolic acid B was 286 nm and the injection volume was 20 μL.

### Identification of active ingredients and prediction of KXF-associated targets

Active ingredients of KXF were retrieved from TCMSP (https://www.tcmsp-e.com/) with the filter conditions of human oral bioavailability (OB) ≥ 30% and drug-likeness (DL) ≥ 0.18. TCMSP was also employed to identify the targets of bioactive molecules. The results of target prediction were sorted from high to low according to “Probability”, and the gene names of the drug targets were retrieved through the UniProt search function in the UniProt database (http://www.uniprot.org/).

### Screening of drug–disease targets

Genecards (https://www.genecards.org/) were used to obtain targets of DCM with a screening criterion of the relevance score > 10. Targets of KXF and targets of DCM were cross-referenced to identify the potential targets of KXF for DCM.

### Visualization of the component-target–disease network and protein–protein interaction network

All intersected targets of the active compounds and disease-related genes were put into the Cytoscape software (Version 3.7.2) to obtain intersections among intersected genes and visualize the ingredient–pathway network. STRING version 11.0 was used to evaluate the PPI information of the overlapped genes, and their biological functions were also obtained with a combine score ≥ 0.4 and hiding the disconnected nodes in the network.

### Functional enrichment analysis of the potential action targets

The Bioconductor database (http://www.bioconductor.org/) was used to query drug–disease target genes. Gene Ontology (GO) enrichment analysis and Kyoto Encyclopedia of Genes and Genomes (KEGG) enrichment analysis were performed for the potential targets of KXF (*p* < 0.05).

### Construction of the medicinal material–component–target–pathway network

The action targets and their related signaling pathways were predicted and then imported into the Cytoscape 3.7.2 software for constructing a network of medicinal material-component-target-pathway to explore the overall pharmacological mechanism of KXF. The topological characteristics of the nodes were evaluated, including degree, betweenness, and closeness. Nodes in the network, connected by edges, represent medicinal materials, ingredients, targets, and pathways. The degree of a node is the number of edges connected to that node, which means that the higher degree, the more nodes it is directly connected to, and the more importance the node has in the network. The closer the nodes are connected, the more influential the nodes are in the network.

### Animals

#### Animal administration

The transgenic zebrafish line (Flila: egfp; Gata1: dsred), which expresses egfp at endothelial cells and dsred at blood cells, was kindly provided by Basic Medical Sciences College of Fudan University. Embryos produced by natural spawning from paired mating were raised in an E3 medium (0.29 g NaCl, 0.0133g KCl, 0.0365 g CaCl_2_, 0.0815 g MgCl_2_·6H_2_O_2_, pH 7.4). All animal experiments were conducted under the standards of national and EU regulations.

#### Toxicity analysis of KXF in zebrafish

Tg (Flila: egfp; Gata1: dsred) of 48hpf transgenic zebrafish was treated with different dosages of KXF for 48 h to determine the safe dosage of KXF by observing the morphology and heart rate of the zebrafish.

#### Model of normal lymphatic vessels in zebrafish

At 48 h post fecundation (hpf), healthy zebrafish embryos were picked out and distributed into a 12-well microplate with 10 fish per well. The embryos were randomly divided into 3 groups including the NC group, the low KXF (15 μg/ml) group, and the high KXF (45 μg/ml) group. The 48 hpf zebrafish embryos were treated with different concentrations (15, 45 μg/ml) of KXF for 48 h. The embryo treated with 0.2% DMSO served as vehicle control. Each group consisted of 30 fish.

#### Model of impaired lymphatic vessels in zebrafish

The 48hpf zebrafish were picked out and divided into 4 groups: the NC group, the model group, the low KXF (15 μg/ml) group, and the high KXF (45 μg/ml) group. The 48 hpf zebrafish embryos were treated with different dosages (15, 45 μg/ml) of KXF for 48 h with a pre-treatment of 30 μM MAZ51 (a selective inhibitor of VEGFR-3(Flt-4) tyrosine kinase) for 6 h. Embryos of the NC group were treated with 0.2% DMSO served as the vehicle control. Each group consisted of 30 fish.

#### Morphological observation and quantification of lymphatic thoracic ducts of zebrafish

After treatment for 48 h of KXF, zebrafish embryos were anesthetized, spread on the coverslip, and fixed horizontally. The images of the zebrafish embryos were obtained by a confocal fluorescence imaging microscope (Olympus). The thoracic duct (TD) is the only lymphatic vessel directly observed in the embryo, which is located between the dorsal aorta (DA) and posterior cardinal vein (PCV). The fluorescence intensity of the thoracic lymphatic vessels was measured from the seventh or eighth to 18th body segments, respectively.

#### Real-time quantitative PCR

The RNA of each group was harvested and isolated by using a Tri reagent, the concentration of which was quantified by a Nano Drop 2000. The purified RNA was reversely transcribed into cDNA using the QuantiNova Reverse Transcription Kit and then amplified in a StepOnePlus PCR machine. The sequences for the primers were as follows: the forward primer sequence of *VEGF-C* is 5′-TCT​CGG​AAT​GTG​TCT​AAC​CGC-3′, the reverse primer sequence of *VEGF-C* is 5′-GTT​TCC​TTC​TTC​ACA​AGC​GGC-3’; the forward primer sequence of *VEGF-A* is 5′-GAC​ATC​AGA​AAA​CGC​GCA​GG-3′, the reverse primer sequence of *VEGF-A* is 5′-TGT​TCA​GTG​TGT​CGT​TAG​CGT-3’; the forward primer sequence of *PROX1* is 5′-AGT​GAA​GAG​GAC​TGT​TGG​GC-3′, the reverse primer sequence of *PROX1* is 5′-ATG​ATG​GTT​GCC​CCT​GGA​AA-3’; the forward primer sequence of *LYVE-1* is 5′-CCC​CCG​CTA​AAT​GAT​GGA​GTT-3’, the reverse primer sequence of *LYVE-1* is 5′-CTC​AAC​CCA​ACC​AAA​CCT​GC-3’; and the forward primer sequence of *EF-1α* is 5′-TAG​ACG​CTA​TCC​TAC​CCC​CCA-3′, the reverse primer sequence of *EF-1α* is 5′-GTG​AAA​TCA​GCA​GCA​CCC​TTG-3’. The results of RT-qPCR were calculated based on the 2-DeltaDeltaCt method.

### Cells

#### The cultivation of lymphatic endothelial cells

Dr. Ran from the University of Illinois (USA) provided the lymphatic endothelial cell (LEC) line. LECs were cultured in a medium consisting of MEM-ALPHA supplemented with 10% fetal bovine serum (FBS) and 1% penicillin–streptomycin in a constant-temperature incubator at 37 C (5% CO_2_). During the logarithmic growth phase of the cells, Trypsin-EDTA (0.25%) was used for trypsinization and subculture. Resuscitated third-generation cells were used for the experiments.

#### Cell proliferation assay

According to the manufacturer’s instructions, the proliferation of LECs was determined by the Cell Counting Kit-8 (CCK-8). The cells were seeded into 96-well plates at the density of 7 × 10^^2^ cells/well and incubated with MEM-ALPHA (2% FBS) in an incubator with an environment of 37°C and 5% CO_2_ for 24 h. After that, the culture medium was replaced with MEM-ALPHA (2% FBS) containing different concentrations of Salvianolic acid B (SAB, the major active ingredient of KXF) (5, 10, 15, 20, 25, 37.5, 50, and 100 μg/ml) and MEM-ALPHA (2% FBS) was as the vehicle control. After 24, 48, and 72 h, 10 μL of the CCK-8 solution was added to each well and incubated for another 2 h. Finally, the optical density (OD) of the cells was measured at 450 nm. The cell viability was calculated as follows:

$$\text{Cell viability}\left(\%\right)=\;\frac{OD\;{\text{value}}_{experimental}\;-OD\;{\text{value}}_{\;blank}}{OD\;{\text{value}}_{control}\;-OD\;{\text{value}}_{blank}}\times 100\text{\%}\text{.}$$

#### Wound healing assay

Four straight lines were drawn on the bottom of the 6-well plate using the sterilized ruler. The cells were plated in a 6-well plate at the density of 4 × 10^^5^cells/well and incubated with MEM-ALPHA (10% FBS) in an incubator with an environment of 37°C and 5% CO_2_. After 24 h, a 200 μl yellow pipette tip was used to draw a scratch perpendicular to the lines on the bottom of the plate, and non-adherent cells were gently washed off with PBS. After that, MEM-ALPHA (1% FBS) containing different concentrations of Salvianolic acid B (5, 10, and 20 μg/ml) was added in the wells, respectively, and MEM-ALPHA (1% FBS) was as the vehicle control. Then, the wound width was recorded using an inverted microscope after 0 and 24 h with a magnification of 4 × , and the formula was as follows:

$$Migration\;rate\left(\%\right)\;=\frac{width\;of\;0h-width\;of\;24h}{width\;of\;0h}\;\times 100\%.$$

#### Western blot analysis

The cells were plated in a 6-well plate at the density of 2 × 10^^5^cells/well and incubated with MEM-ALPHA (10% FBS) in an incubator with an environment of 37°C and 5% CO_2_; after incubating for 24 h, MEM-ALPHA (10% FBS) containing different concentrations of SAB (0, 5, 10, and 20 μg/ml) was added in the wells respectively and incubated for 24 h.

The cells were plated in a 6-well plate at the density of 2 × 10^^5^cells/well and incubated with MEM-ALPHA (10% FBS) in an incubator with an environment of 37°C and 5% CO_2_; After 24 h, MEM-ALPHA (10% FBS) containing different concentrations of Salvianolic acid B (0, 5, 10, 20 μg/ml) and MAZ51 (7.5 μM) were added in the wells respectively and incubated for 24 h, and MEM-ALPHA (10% FBS) without Salvianolic acid B and MAZ51 was as a vehicle.

After the cells were harvested and lysed, the samples (30 μg protein/lane) were fractionated by PAGE, transferred to PVDF membranes, blocked by a blocking buffer, and incubated with antibodies to VEGF-C, FLT4/VEGFR-3, and GAPDH at a dilution of 1:1,000 overnight at 4°C. The membrane was washed three times with a Tris buffered saline with Tween 20 (TBST) for 15 min and incubated with HRP-linked anti-rabbit IgG for 1 h at room temperature. The strips were visualized using BeyoECL Plus. GAPDH was used as an internal control for normalization. All the experiments were done thrice.

### Statistical analysis

Quantitative data were presented as mean ± SD $\left(\bar{x}\pm s\right)$ . A one-way analysis of variance (ANOVA) was subjected for comparison between more than two groups, and the LSD-t test was used for comparisons between groups. All analyses were performed using the GraphPad Prism version 8.0. A value of *p* < 0.05 was considered to be significant.

## Results

### High-performance liquid chromatography

**FIGURE 1:**
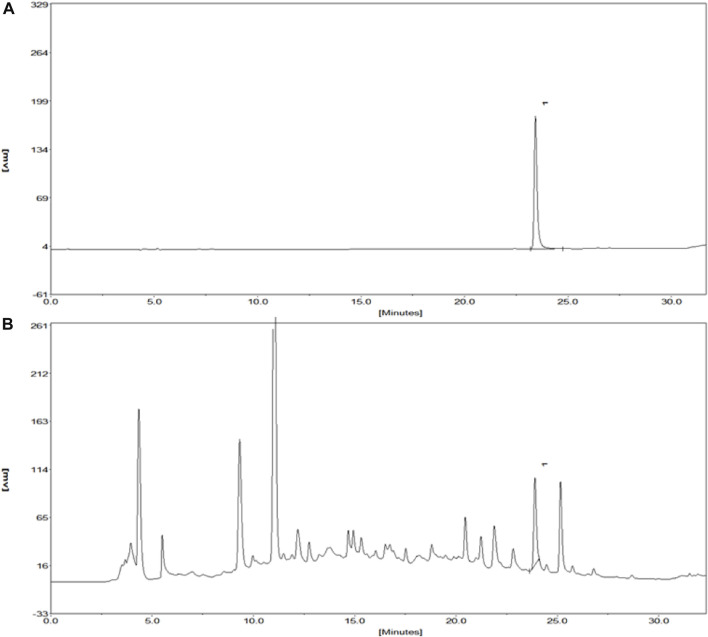
Chromatograms of the Salvianolic acid B standard and extract of KXF. **(A)**. Chromatogram of the Salvianolic acid B standard at 286 nm. The retention time of the Salvianolic acid B standard was 23.42 min, and the peak height and peak area of which were 178.11 and 1785.6 mV*sec, respectively. **(B).** Chromatogram of the extract of KXF at 286 nm. (1). Salvianolic acid B.

Kuoxin Decoction is a traditional Chinese medicine compound, which contains a variety of chemical ingredients, and Salvianolic acid B, Calycosin, and 5-hydroxymethylfurfural are major chemical elements of KXF. Therefore, a HPLC analysis was used to determine the contents of these components. As shown in Figure 1A and Supplementary Table S3, the retention time of Salvianolic acid B standard at 286 nm was 23.42 min, and the peak height and area were 178.11 and 1785.6 mV*sec, respectively. The content of Salvianolic acid B in the KXF extract was 0.2085 %, and the retention time, peak height, and peak area were 23.92 min, 88.13 mV, and 773.71 mV*sec, respectively (Figure 1B and Supplementary Table S3). Our previous studies have confirmed that Calycosin and 5-hydroxymethylfurfural did not significantly promote the proliferation of lymphatic endothelial cells, so the chromatograms of these ingredients have not been added in this article.

### Identification of active ingredients and prediction of Kuoxin Decoction-associated targets

**FIGURE 2:**
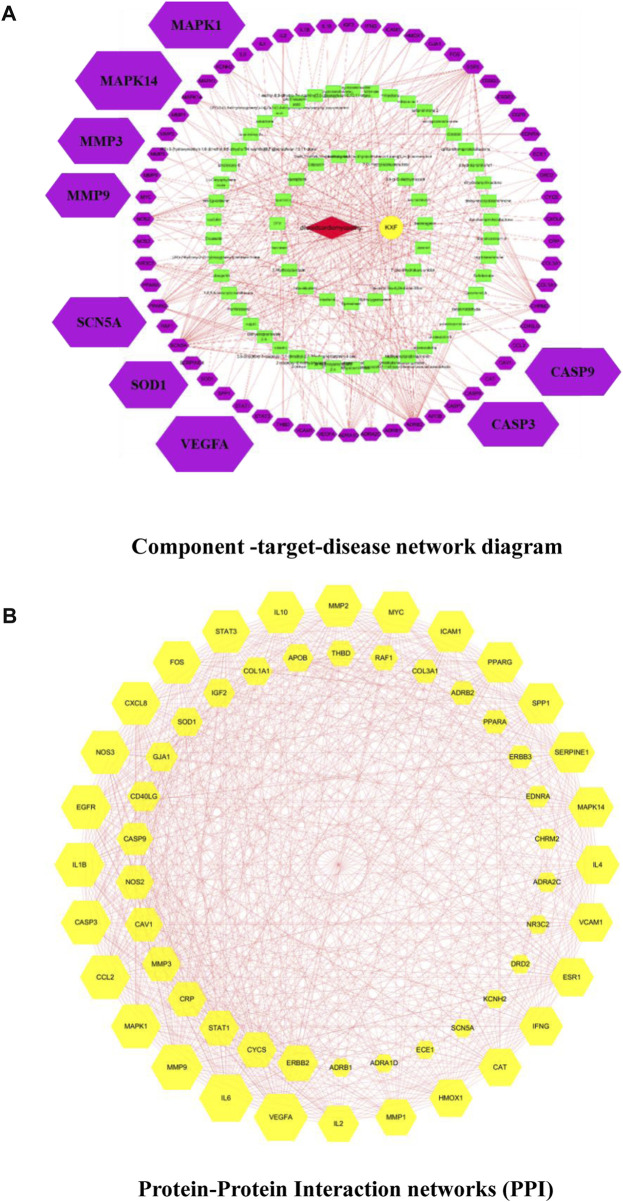
**(A)**. Component-target–disease network diagram. Cross-references to KXF and DCM targets to identify potential targets of KXF associated with DCM. Red nodes represent DCM. The yellow node represents KXF. Green nodes represent the compounds in KXF. Purple nodes represent potential targets of KXF of DCM. **(B)**. Protein–protein interaction network (PPI) shows the interactions between overlapping genes. The yellow node represents a gene, and the red line represents the interaction between genes. The larger the area covered by a gene, the more pronounced the effect of KXF has on that gene.

We used the TCMSP database to identify 106 active ingredients and 224 corresponding targets from KXF (Supplementary Table S2). We obtained 722 DCM-relevant genes after administering the search and queries in the GeneCards database (Supplementary Table S2). Furthermore, an intersection between ingredient-targeted and DCM-relevant genes was performed, and 58 overlapped genes were obtained eventually. The chemical component-target–disease network is shown in Figure 2A.

Based on the overlapped genes, we used the Cytoscape 3.7.2 software to construct a PPI network with a combined_score ≥ 0.4 (Figure 2B). According to the degree value, genes in the top 20 are *VEGF-A*, *IL6*, *MMP9*, *MAPK1*, *CCL2*, *CASP3*, *IL1B*, *EGFR*, *NOS3*, *CXCL8*, *FOS*, *STAT3*, *IL10*, *MMP2*, *MYC*, *ICAM1*, *PPARG*, *SPP1*, *SERPINE1*, and *MAPK14* (Supplementary Table S2). Notably, it was confirmed that *STAT1/STAT3*, *IL1B/IL2/IL4/IL6/CXCL8/IL10*, *CCL2* (Aldrich and Sevick-Muraca, 2013; Nielsen et al., 2013; Lim et al., 2016; Korbecki et al., 2021), *EGFR* (Gore et al., 2016), *ERBB2/ERBB3*, *FOS* (Ming et al., 2009), *VCAM1* (Prangsaengtong et al., 2018a), *ICAM1* (García-Silva et al., 2021), and *IGF2* (Björndahl et al., 2005; Lu et al., 2009) were related to lymphangiogenesis.

### GO and KEGG enrichment analysis

**FIGURE 3:**
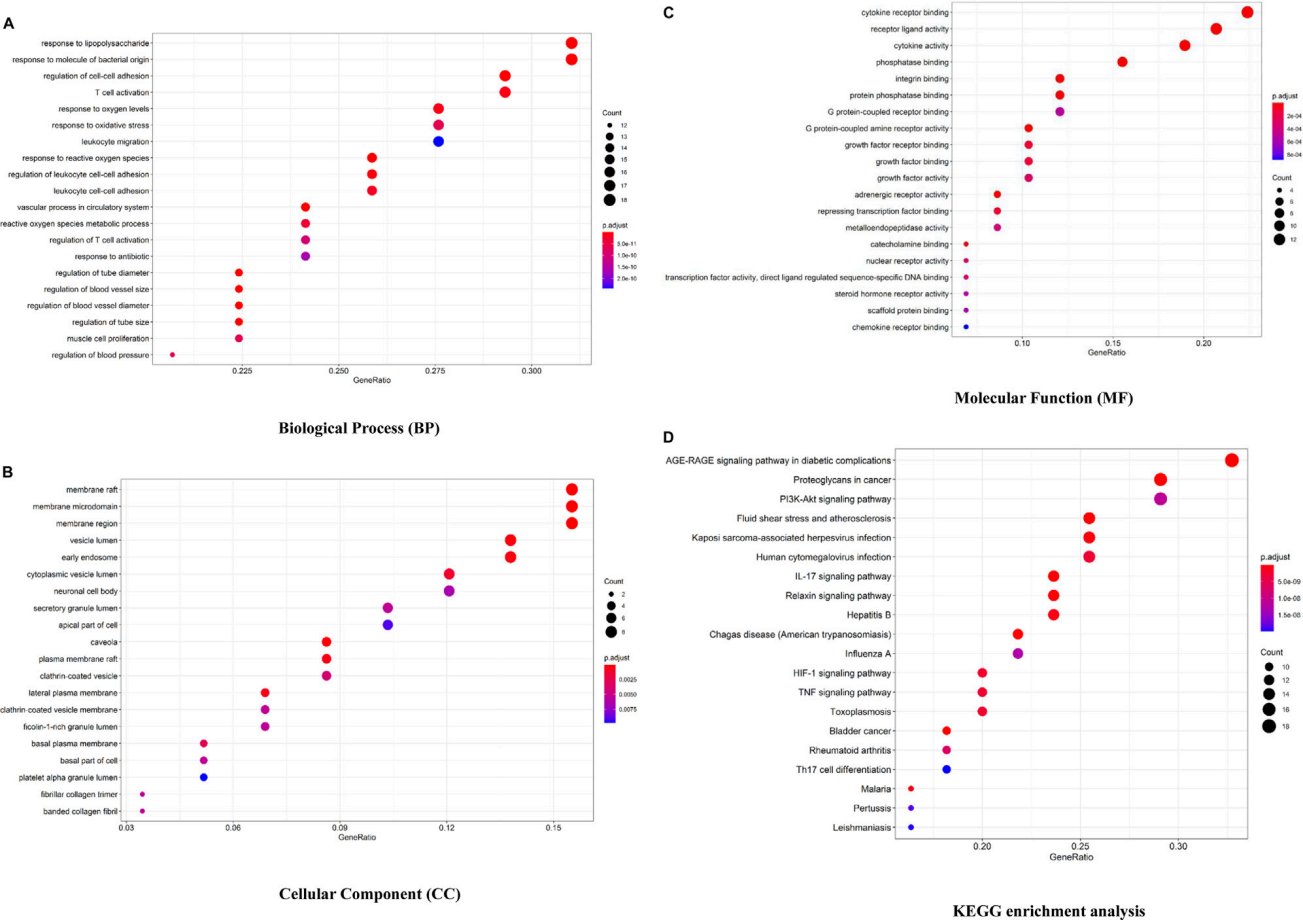
Bioconductor database-enriched pathways and GO entries. **(A)**. GO enrichment entries in the top 20 about the biological process (BP). **(B)**. GO enrichment entries in the top 20 about cellular component (CC). **(C)**. GO enrichment entries in the top 20 about molecular function (MF). **(D)**. KEGG enrichment entries in the top 20 (*p* < 0.05). The color of the bubble represents the value of *p,* and the size of the bubble represents the count of relative entries.

The Bioconductor database was used to perform a GO enrichment analysis of 58 overlapped genes. The results of the GO analysis are shown in Figures 3A–C and Supplementary Table S2. There are 1,577 entries in the biological process, mainly involving the response to lipopolysaccharide, angiogenesis, regulation of cell–cell adhesion, regulation of the inflammatory response, etc. For example, Ghose S (Ghose et al., 2015) confirmed that δ-Catenin (an adherens junction protein) promotes lymphangiogenesis, and Yang Y (Yang et al., 2015) found that cell adhesion mediated by VCAM-ITGα9 interactions was related to lymphatic development. Lymphatics play an essential pathophysiological role in promoting fluid and immune cell tissue clearance, and different immune cell populations impact lymphatic remodeling exerting either stimulatory or inhibitory effects on the lymphatic endothelial cell growth and survival (Angeli et al., 2006; Kataru et al., 2011; Förster et al., 2012).

In the part of a cellular component analysis, 57 items were obtained mainly concentrated in the membrane raft, membrane microdomain, membrane area, etc. There are 77 items of molecular function, which are mainly related to the cytokine receptor binding function, receptor–ligand activity, and cytokine activity, and many cytokines and their receptors are associated with lymphangiogenesis, like interleukin 7/interleukin 7 receptor and thymic stromal lymphopoietin (Ming et al., 2012; Braile et al., 2021).

As shown in Figure 3D and Supplementary Table S2, 132 signaling pathways were identified through the KEGG enrichment analysis (*p* < 0.05), including AGE-RAGE, PI3K-AKT (Roy et al., 2020), IL-17 (Chauhan et al., 2011; Chen et al., 2010: Park et al., 2018), HIF-1 (Prangsaengtong et al., 2018b; Roy et al., 2020), and TNF (Hong et al., 2016; Schwager and Detmar, 2019) signaling pathways associated with lymphangiogenesis.

### Construction of medicinal material–chemical component–target–pathway network

**FIGURE 4:**
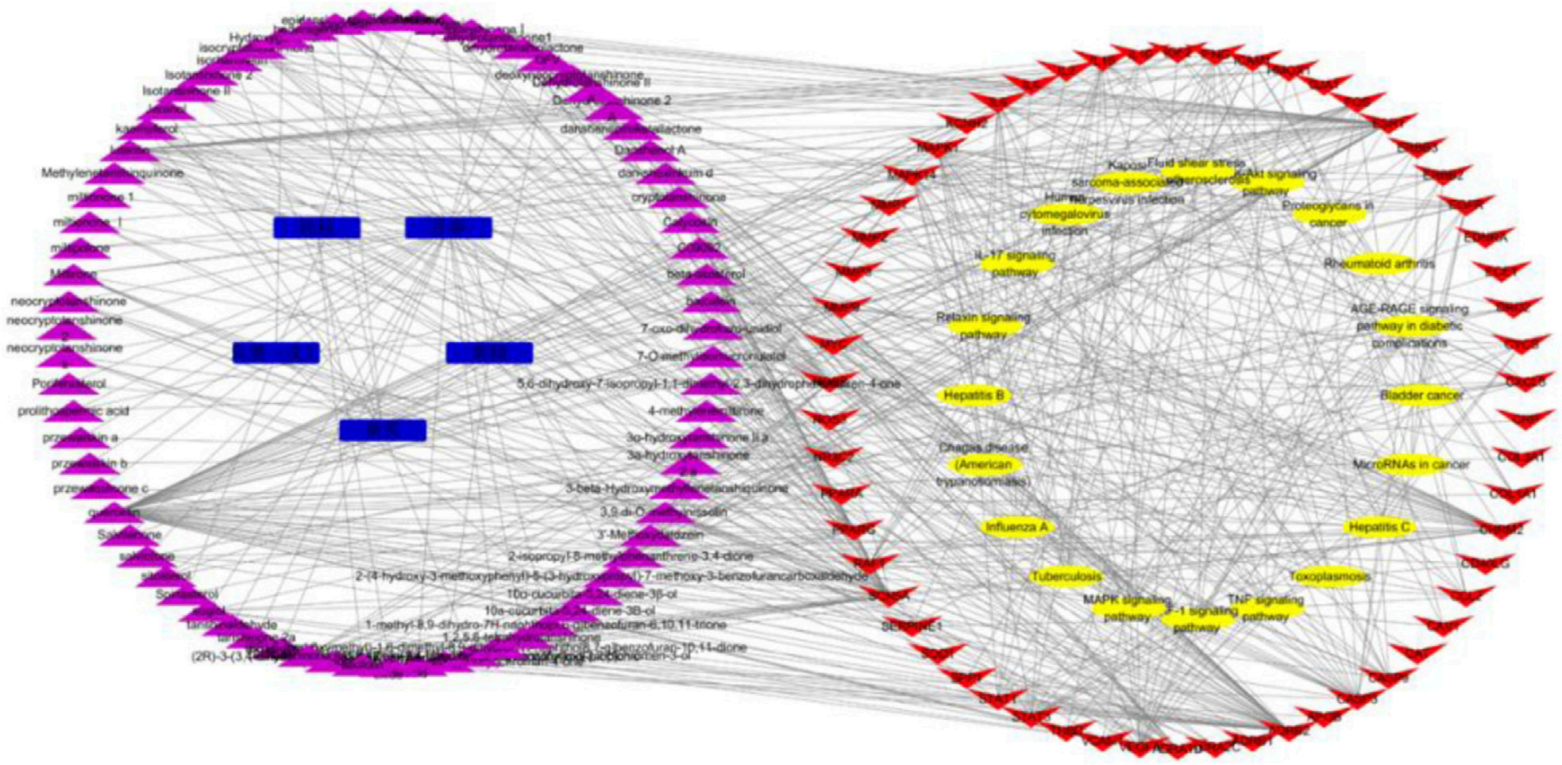
Medicinal material-component-target-signal pathway network. Blue nodes represent the 5 principal components of KXF, purple nodes represent active compounds, red nodes represent relative genes, and yellow nodes represent signaling pathways. 黄芪:*Astragalus mongholicus* Bunge (Huangqi), 黄精:*Polygonatum kingianum* Collett and Hemsl (Huangjing), 丹参:*Salvia miltiorrhiza* Bunge (Danshen), 桂枝:*Neolitsea cassia* (L.) Kosterm (Guizhi), 瓜蒌(皮):*Trichosanthes kirilowii* Maxim (Gualoupi).

The action targets and related signaling pathways from KXF were imported into the Cytoscape software to construct compound–target networks and compound–target pathway networks to explore the overall pharmacological mechanisms of KXF. As shown in Figure 4, the network is composed of 157 nodes, including 5 medicines, 74 active ingredients, 58 related genes, and 20 signaling pathways.

### Toxicity analysis of Kuoxin Decoction in zebrafish

**FIGURE 5:**
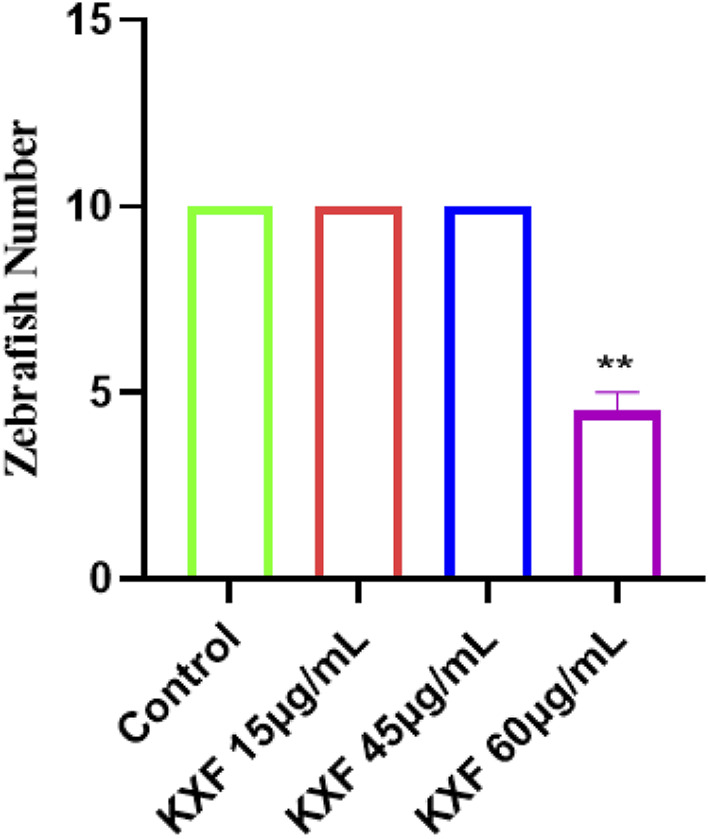
Toxicity analysis of KXF in zebrafish. Tg (Flila: egfp; Gata1: dsred) transgenic zebrafish of 48hpf was treated with different dosages of KXF for 48 h to determine the safe dosage of KXF. Quantitation of the survival zebrafish number. **p < 0.01 vs. control group.

Tg (Flila: egfp; Gata1: dsred) of 48hpf transgenic zebrafish were treated with different dosages of KXF for 48 h to determine the safe dosage of KXF. As shown in Table 1 and Figure 5, the results showed that the morphology and heart rate was normal in zebrafish when the KXF concentration was less than or equal to 45 μg/ml compared with the control group. However, when the concentration of KXF was 60 μg/ml, the phenomena of body deformity and disappearing heartbeat occurred in the zebrafish (*p* < 0.01), which indicated that the concentrations of KXF ( ≤45μg/mL) had no obvious toxic effect on the growth and development of zebrafish, so we chose the dosage of 15, 45 μg/ml in the following experiments.

**TABLE 1:** Toxicity analysis of KXF in zebrafish.

Dosage of KXF (μg/ml)	Number of alive zebrafish $\left(\bar{x}\pm s\right)$
0	10 ± 0
15	10 ± 0
45	10 ± 0
60	4.5 ± 0.5

### Kuoxin Decoction promotes lymphangiogenesis in a normal zebrafish model

**FIGURE 6:**
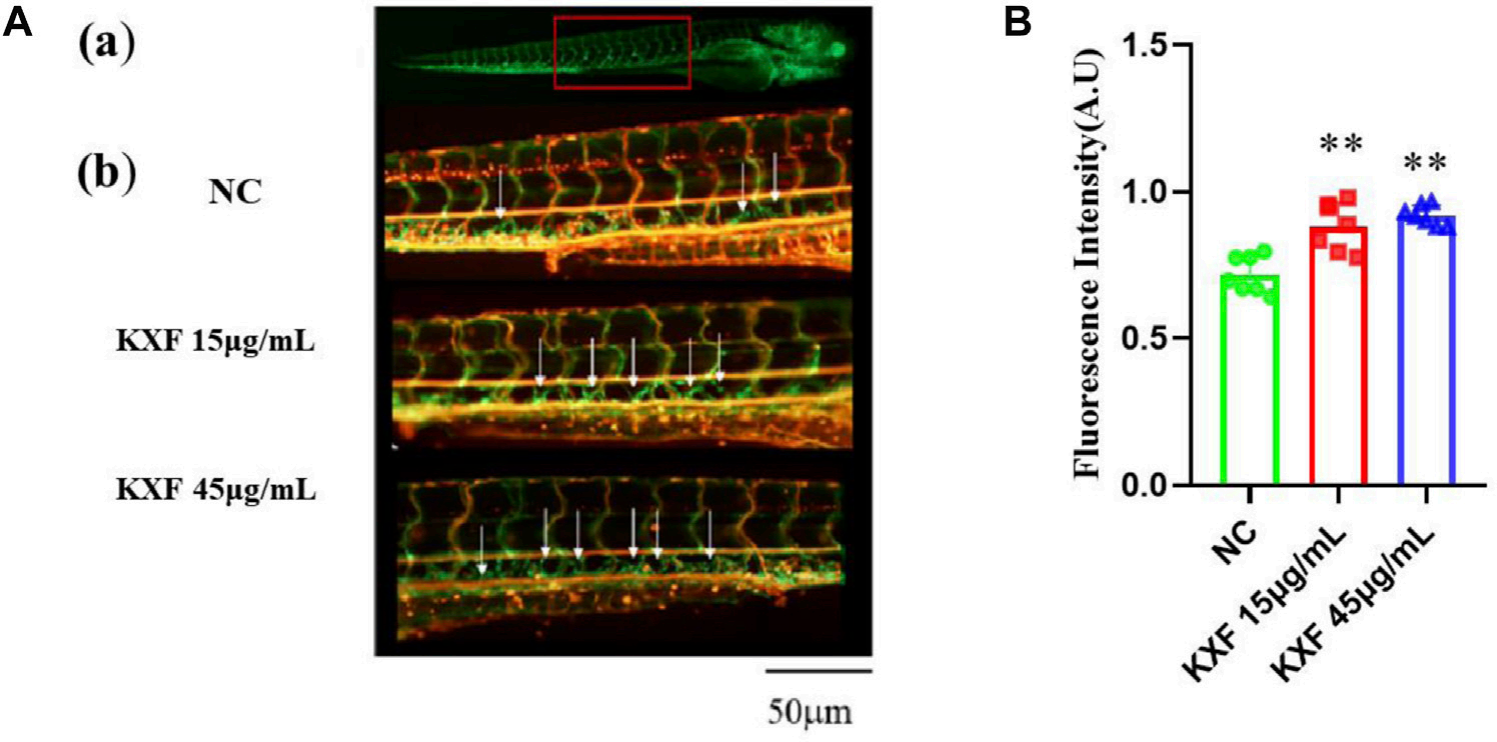
KXF increased the lymphatic thoracic duct formation in zebrafish. **(A)**. Confocal image of the 96 hpf. zebrafish vascular system. **(A)**. The red-boxed region indicates the area of the confocal image (from the seventh or eighth to the 18th somite). **(B)**. Representative confocal images show that KXF promoted the thoracic lymphatic duct formation in zebrafish, the white arrow indicates the thoracic lymphatic duct; **(B)**. Quantitation of the fluorescence intensity of the thoracic lymphatic duct. **p < 0.01 vs. NC group.

To determine the effect of KXF on lymphatic vessels, we used a zebrafish screening system. At 48hpf, the zebrafish were treated with KXF (15, 45 μg/ml) for 48 h, and we found that, 15 and 45 μg/ml KXF significantly increased the fluorescence intensity in the thoracic duct compared to the NC group. (*p* < 0.01) (Figure 6).

### Kuoxin Decoction promotes lymphangiogenesis in the impaired lymphatic thoracic duct induced by the VEGFR-3 kinase inhibitor (MAZ51)

**FIGURE 7:**
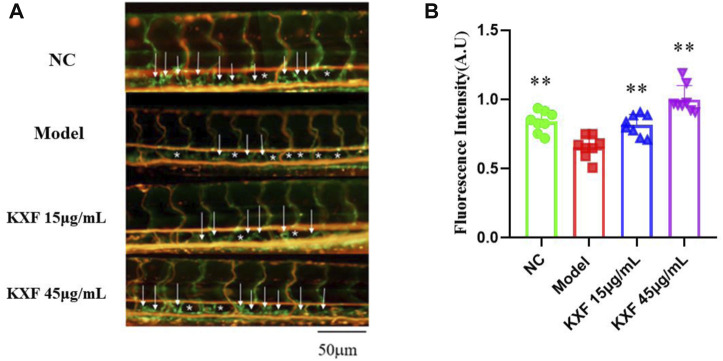
Impaired lymphatic thoracic duct formation induced by VEGFR-3 kinase inhibitor (MAZ51) was rescued by KXF. **(A)**. Representative confocal images show that KXF promoted the thoracic lymphatic duct formation in zebrafish. The white arrow indicates the thoracic lymphatic duct, and the white star indicates a lack of lymphatic vessels; **(B)**. Quantitation of the fluorescence intensity of the thoracic lymphatic duct. * *p < 0.01 vs. model group.

Tg (Flila: EGFP; Gata1: DsRed) transgenic zebrafish embryos of 48hpf were pretreated with 30 μM vascular endothelial growth factors receptor-3 (VEGFR-3) kinase inhibitor (MAZ51) for 6 h, and then treated with different concentrations of KXF (15,45 μg/ml) for 48 h. We found that MAZ51 remarkably impaired the thoracic duct formation (*p* < 0.01), but treatment with KXF (15, 45 μg/ml) significantly improved the damage to the thoracic duct (*p* < 0.01) (Figure 7).

### Kuoxin Decoction promotes the expression of *VEGF-C* and *VEGF-A* mRNA in two models of zebrafish

**FIGURE 8:**
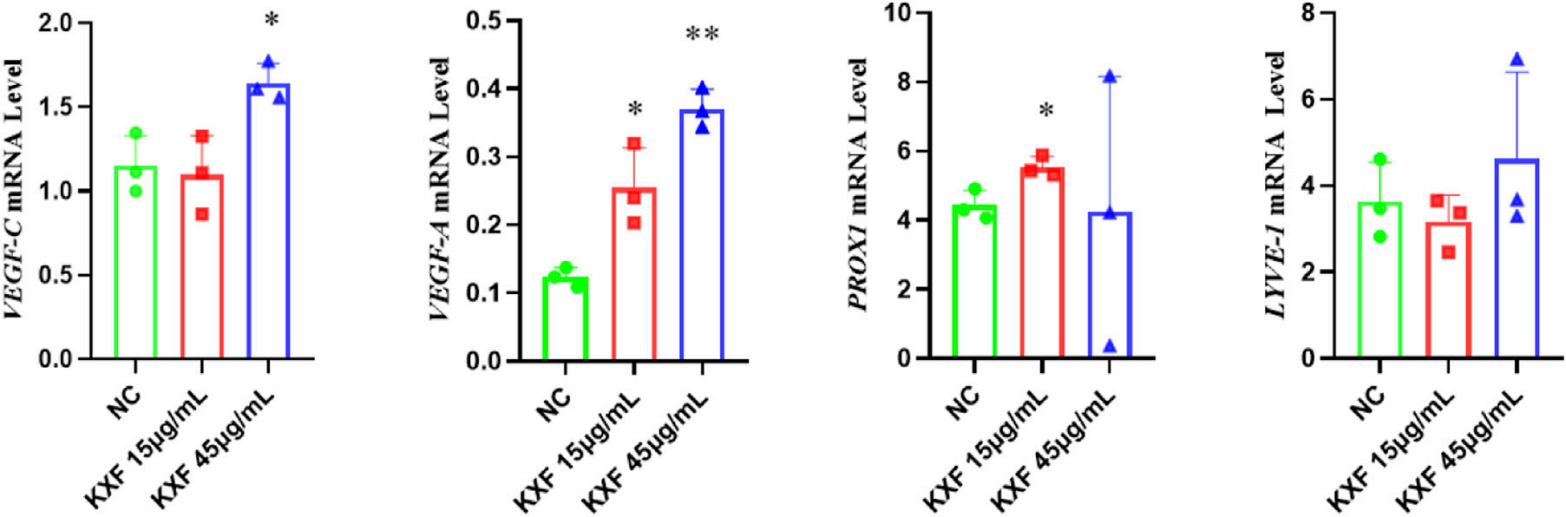
KXF promoted the expression of mRNAs related to the lymphatic vessel. The image represents the expression levels of *VEGF-C*, *VEGF-A*, *PROX1*, and *LYVE-1* mRNA in normal zebrafish with the treatment of two dosages of KXF for 48 h. *p < 0.05 vs. NC group, **p < 0.01 vs. NC group.

As shown in Figure 8, *VEGF-C*, *VEGF-A*, and *PROX1* mRNA levels were significantly increased in the KXF-treated group compared with the NC group (*p* < 0.05), while the *LYVE-1* mRNA levels had no significant difference between the NC group and other groups (*p* > 0.05).

**FIGURE 9:**
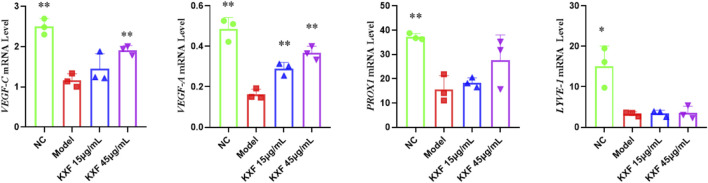
KXF increased the expression of mRNAs associated with lymphatic vessels after zebrafish thoracic lymphatic vessels were damaged by the VEGFR-3 kinase inhibitor (MAZ51). After the pre-treatment of MAZ51 (6 h), the expression levels of *VEGF-C*, *VEGF-A*, *PROX1*, and *LYVE-1* mRNA in impaired zebrafish with two dosages of KXF for 48 h. *p < 0.05 vs. Model group, **p < 0.01 vs. Model group.

As shown in Figure 9, *VEGF-C*(*p* < 0.01), *VEGF-A* (*p* < 0.01), *PROX1* (*p* < 0.01), and *LYVE-1*(*p* < 0.05) mRNA levels were significantly decreased in the model group compared with the NC group. In contrast, *VEGF-C* and *VEGF-A* mRNA levels were increased after KXF treatment compared with the model group (*p* < 0.01). These results indicated that KXF could promote lymphangiogenesis by upregulating the expressions of *VEGF-C* and *VEGF-A* mRNA.

### Salvianolic acid B (the major active ingredient of Kuoxin decoction) promotes the proliferation of lymphatic endothelial cells

**FIGURE 10:**
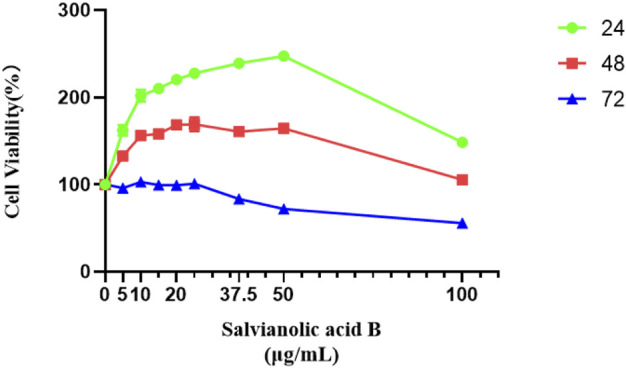
Salvianolic acid B promoted the proliferation of lymphatic endothelial cells. The image represents the viability of lymphatic endothelial cells treated with different dosages of Salvianolic acid B (5, 10, 15, 20, 25, 37.5, 50, and 100 μg/ml). The green curve represents the cell viability after 24 h of treatment with different concentrations of SAB. The red curve represents the cell viability after 48 h of treatment with different concentrations of SAB. The blue curve represents the cell viability after 72 h of treatment with different concentrations of SAB.

As shown in Figure 10, all groups treated with Salvianolic acid B for 24 h could improve the survival rate of LECs compared with the control group (*p* < 0.05). When treatment time was extended to 48 h, the survival rate of the Salvianolic acid B-treated group was higher than that of the control group except for the 100 μg/ml group (*p* < 0.05); although there was a tendency to promote proliferation in the 100 μg/ml group, the difference was not statistically significant when compared with the control group (*p* > 0.05). The cell survival rate was significantly lower than that in the control group when the concentration of SAB was 37.5, 50, and 100 μg/ml when the treatment time was 72 h (*p* < 0.05). These results showed that Salvianolic acid B could promote the proliferation of LECs in the concentration range of 5–25 μg/ml, and the optimal incubation time was 24 h. The effect curve peaked at a concentration of 25 μg/ml when the incubation time was 48 and 72 h, respectively. Considering the stability of drug action, the doses of Salvianolic acid B were 5, 10, and 20 μg/ml, and 24 h was chosen as the incubation time in the subsequent experiments.

### Salvianolic acid B promotes the migration of lymphatic endothelial cells

**FIGURE 11:**
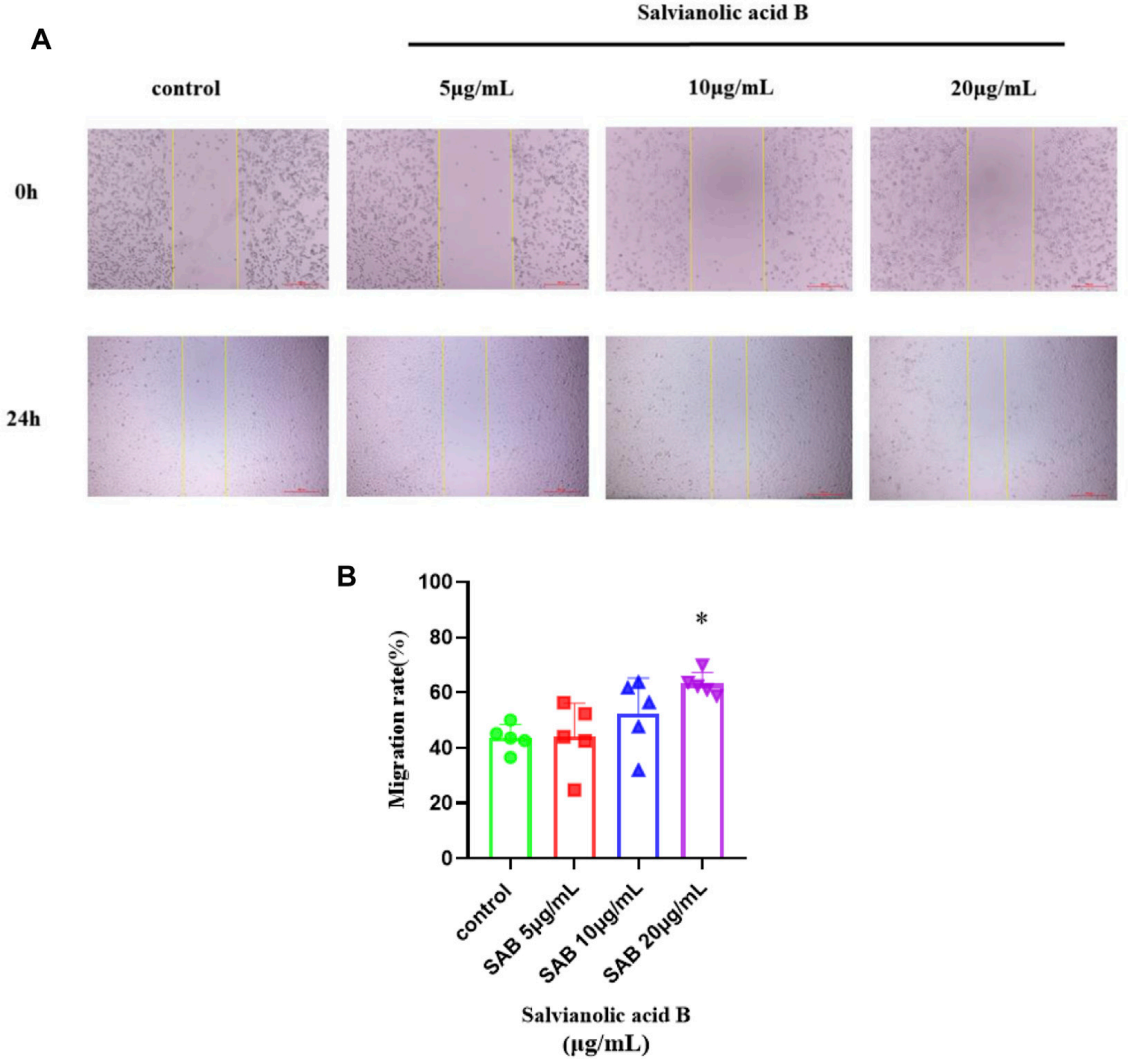
Salvianolic acid B promoted the migration of lymphatic endothelial cells. The image represents the changes in the scratch width after Salvianolic acid B treatment. **(A)**. The images of the scratches before LECs were treated and after LECs were treated with three dosages of SAB (5, 10, and 20 μg/ml) for 24 h. The distance between two yellow lines is the width of the scratch. **(B)**. Quantitation of migration rate. *p < 0.05 vs. Control group.

As shown in Figure 11, the migration rate of Salvianolic acid B at doses of 10 and 20 μg/ml was higher than that of the control group, and the effect of 20 μg/ml was statistically significant (*p* < 0.05). There was no significant difference between the 5 μg/ml group and the control group (*p* > 0.05). These results imply that Salvianolic acid B promotes the migration of lymphatic endothelial cells.

### Salvianolic acid B promotes the protein expression of VEGF-C and VEGFR-3 in normal lymphatic endothelial cells

**FIGURE 12:**
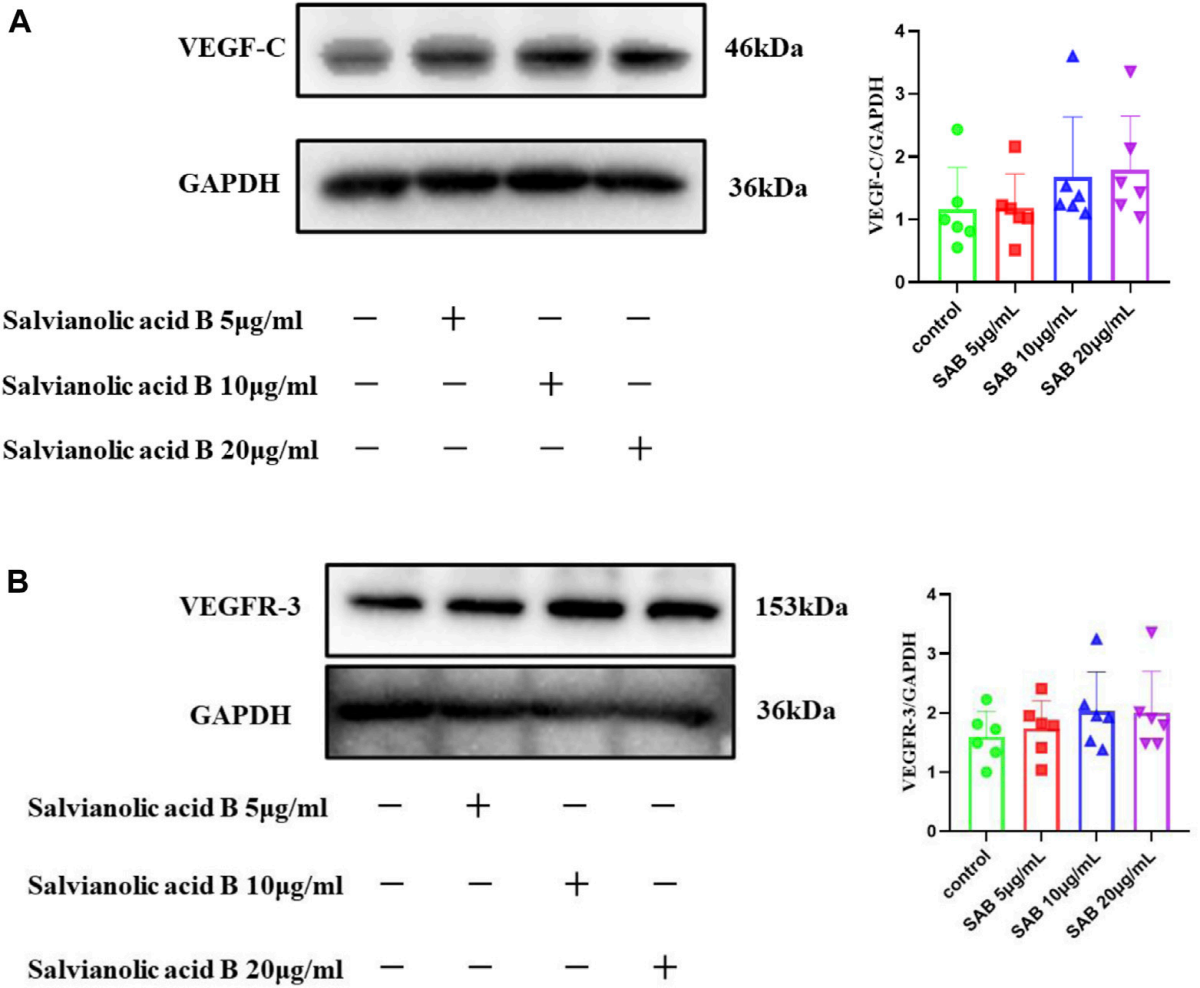
The protein expressions of VEGF-C and VEGFR-3. LECs were treated with different concentrations of SAB for 24h, and cell lysates were prepared and analyzed by western blot assay. **(A)**. Quantitation of the protein expression of VEGF-C. **(B)**. Quantitation of the protein expression of VEGFR-3 protein.

As shown in Figure 12, there was a trend that the three doses of Salvianolic acid B promoted the protein expression of VEGF-C and VEGFR-3. Still, there was no statistical difference compared with the control group (*p* > 0.05).

### Salvianolic acid B promotes the protein expression of VEGF-C and VEGFR-3 in lymphatic endothelial cells under injury

**FIGURE 13:**
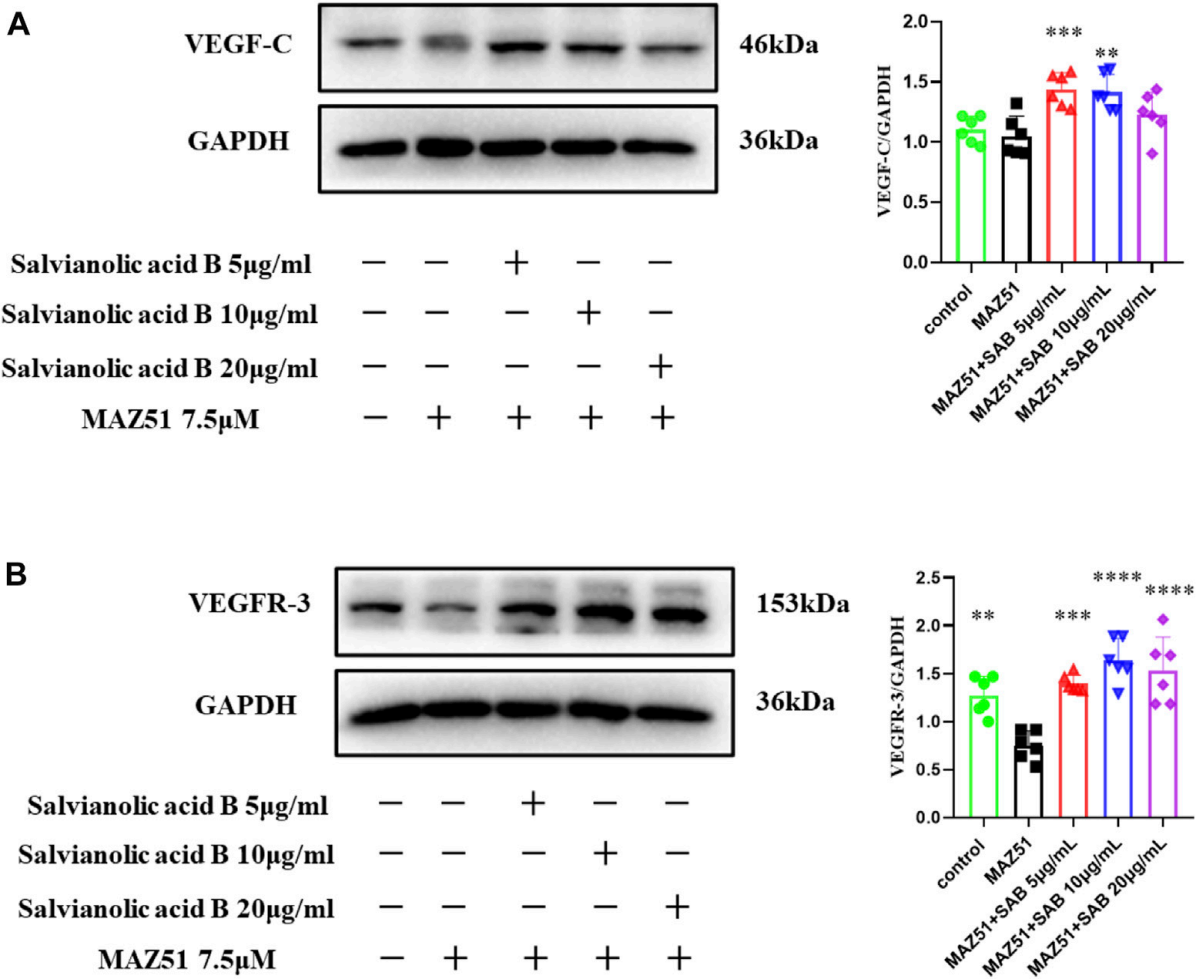
Salvianolic acid B promoted the protein expressions of VEGF-C and VEGFR-3 proteins in lymphatic endothelial cells under injury. After the LECs were treated with MAZ51 for 24 h to induce injury, along with the treatment of three dosages of SAB for 24 h, cell lysates were prepared and analyzed by western blot assay. **(A)**. Quantitation of the protein expression of VEGF-C. **(B)**. Quantitation of protein expression of VEGFR-3. **p < 0.01 vs. MAZ51 group; ***p < 0.001 vs. MAZ51 group; ****p < 0.0001 vs. MAZ51 group.

Lymphatic endothelial cells were simultaneously treated by MAZ51 and three doses of Salvianolic acid B (5, 10, 20 μg/ml) for 24 h, respectively. As shown in Figure 13B, the VEGFR-3 protein expression level in the MAZ51-only group was significantly lower than that in the control group (*p* < 0.01). Compared with the group treated with MAZ51 alone, the expression of VEGFR-3 was significantly increased after co-culturing with three doses of Salvianolic acid B. The optimal effective dose was 10 μg/ml (*p* < 0.0001), which indicated that Salvianolic acid B promoted the protein expression of VEGFR-3 in LECs after injury. As shown in Figure 13A, no significant differences were found in the VEGF-C levels between the MAZ51 group and the Control group (*p* > 0.05); however, the VEGF-C protein expression was significantly increased after the co-culture with Salvianolic acid B at 5 and 10 μg/ml, respectively (*p* < 0.001, *p* < 0.01). Salvianolic acid B (20 μg/ml) showed an increasing trend of the VEGF-C expression, but there was no statistical difference when compared with the MAZ51 group (*p* > 0.05). These results suggest that Salvianolic acid B promotes the protein expressions of VEGF-C and VEGFR-3 in LECs after injury.

## Discussion

Kuoxin Decoction (Wang et al., 2017) is an herbal formulation for the treatment of dilated cardiomyopathy, which significantly improves patients’ cardiac function and clinical symptoms. In the present study, 106 active compounds of KXF and 224 genes were identified from the TCMSP database, and there were 722 disease-related targets (Supplementary Table S2). Cross-referencing the disease targets with compound targets led to 58 overlapping targets. An enrichment analysis was performed on overlapping target genes to evaluate the relationship between these active compounds and DCM.

GO entry and the KEGG pathway revealed a total of 132 signaling pathways, of which, PI3K-AKT (Roy et al., 2020), IL-17 (Chen et al., 2010; Chauhan et al., 2011; Park et al., 2018), HIF-1 (Prangsaengtong et al., 2018a; Roy et al., 2020), and TNF (Hong et al., 2016; Schwager and Detmar, 2019) signaling pathways are associated with lymphangiogenesis. The top 20 targets in the PPI network are as follows, *VEGF-A*, *IL6*, *MMP9*, *MAPK1*, *CCL2*, *CASP3*, *IL1B*, *EGFR*, *NOS3*, *CXCL8*, *FOS*, *STAT3*, *IL10*, *MMP2*, *MYC*, *ICAM1*, *PPARG*, *SPP1*, *SERPINE1*, and *MAPK14*. Most importantly, many research studies have confirmed that *STAT1/STAT3*, *IL1B/IL2/IL4/IL6/CXCL8/IL10*, and *CCL2* (Aldrich and Sevick-Muraca, 2013; Nielsen et al., 2013; Lim et al., 2016; Korbecki et al., 2021), *EGFR* (Gore et al., 2016), FOS (Ming et al., 2009), *VCAM1* (Prangsaengtong et al., 2018b), *ICAM1* (García-Silva et al., 2021), and *IGF2* (Björndahl et al., 2005; Lu et al., 2009) could regulate lymphangiogenesis.

To validate the result of the network analysis, the zebrafish model was further used to observe changes in lymphatic vessels and the expression levels of lymphatic-specific markers such as *VEGF-C*, *PROX1*, and *LYVE-1* mRNA. Ratajska (Ratajska et al., 2014) found that first LYVE-1-bearing cells and tubules occurred on the dorsal atrioventricular sulcus and subsequently these structures gained the Prox-1 antigen in mouse fetuses, which means that there is a certain correlation between the development of cardiac lymphatics and cardiac veins, so, we further analyzed the expression of *VEGF-A* (a key regulator of angiogenesis) to research the mechanisms and targets of KXF in regulating lymphangiogenesis.

Zebrafish are widely used in research studies about cardiovascular diseases because of their 87% genetic similarity to humans and the advantages of an easy observation of blood vessels and lymphatic vessels, fast reproduction, and high rearing density. The transgenic zebrafish line (Flila: egfp; Gata1: dsred), which expresses egfp (green) at the endothelial cells and dsred (red) at the blood cells, was used to study the effect of KXF on lymphangiogenesis of the thoracic duct in zebrafish with or without injury induced by MAZ51. In zebrafish with or without impaired lymphatic vessels, KXF could promote lymphangiogenesis in both models. The results of a RT-qPCR confirmed that the process was accompanied by upregulating the *VEGF-C* and *VEGF-A* mRNA levels.

Many types of research have confirmed that myocardial infarction and atherosclerosis are associated with morphological or functional defects in the lymphatics (Angeli and Harvey, 2015; Henri et al., 2016; Feng et al., 2022). Salvianolic acid B, the major active ingredient of *Salvia miltiorrhiza* Bunge in KXF, has a cardioprotective effect on acute myocardial infarction and atherosclerosis by inhibiting apoptosis and inflammation (Lin et al., 2016; Yang et al., 2020). Thus, we hypothesized that Salvianolic acid B had the effect of regulating lymphangiogenesis and chose Salvianolic acid B for further investigation in LECs.

Lymphatic endothelial cell experiments further confirmed that Salvianolic acid B, one of the active ingredients of KXF, promoted the proliferation and migration of LECs. Meanwhile, Salvianolic acid B could promote the protein expressions of VEGF-C and VEGFR-3. It is consistent with the results of the network analysis, suggesting that KXF promotes lymphangiogenesis by regulating the VEGF family and that the main component exerting this effect may be Salvianolic acid B.

Many studies have shown that the *VEGFR-3*-mediated signaling pathway is crucial in regulating lymphangiogenesis. *VEGFR-3*, a typical lymphoid receptor tyrosine kinase, is mainly expressed in lymphatic endothelial cells, which induces lymphatic vessel germination and affects the development of lymphatic vessels by interacting with VEGF-C or VEGF-D directly. Thus, VEGF-C/VEGFR-3 signaling is critical for LEC proliferation, migration, and survival (Nurmi et al., 2015; Hsu et al., 2019).

*PROX1* (Wu et al., 2014) is a major regulator of lymphatic endothelial cell differentiation, which induces the development of lymphatic progenitor cells and determines the proliferative phenotype of lymphatic endothelial cells. The study of Del Giacco (Del Giacco et al., 2010) demonstrated that the absence of the *PROX1b* activity severely hampers the formation of the thoracic duct in zebrafish. Therefore, it is a highly specific and sensitive marker for LECs. *LYVE-1* (Tang and Zhao, 2015) is a hyaluronic acid receptor on the surface of lymphatic endothelial cells, which can be used to defect LECs as a marker of lymphatic endothelial cells alone or in combination with *PROX1* (Zhang et al., 2017). Podoplanin (Carrasco-Ramírez et al., 2016) is mainly related to the generation of LECs. *PROX1*, *LYVE-1*, and Podoplanin are representative molecules of the VEGF-C/D-VEGFR-3/Nrp2 axis pathway (Wang et al., 2016).

Lymphangiogenesis occurs in adult tissues during inflammation, wound healing, and tumor metastasis. Inflammatory insults are induced by macrophages and granulocytes, and many immune cell populations impact lymphatic remodeling (Angeli et al., 2006; Kataru et al., 2011; Förster et al., 2012). The LECs in afferent lymphatic vessels attract activated dendritic, T, and B cells expressing the chemokine receptor CCR7 by producing its ligand CCL21, which is a secondary lymphoid chemokine (Förster et al., 2008).

Sphingosine-1-phosphate (S1P) is a bioactive lipid, synthesized by sphingosine kinases, that is involved in the paracrine signaling of inflammatory cells. S1P stimulates lymphangiogenesis and regulates lymphatic vessel maturation (Pham et al., 2010).

Nuclear factor (NF-κb) -VEGF-C pathway participates in the lymphangiogenesis of gallbladder carcinoma, and VEGF-C is activated in response to proinflammatory cytokines such as TNF-α (Li et al., 2018). IGF-1 is related to lymphangiogenesis, and Chen found that LncCCLM could reduce lymphangiogenesis and lymphatic metastasis in cervical cancer by accelerating the degradation of *IGF-1* mRNA (Chen et al., 2021).

We have verified that SAB could induce the VEGF-C/VEGFR-3 expression, but we did not study how SAB regulates VEGF-C/VEGFR-3 in this study. We will continue to explore the mechanism in our future work, and TNF-α- NF-κb-VEGF-C may be a potential pathway for regulating lymphangiogenesis.

Our previous studies found that the markers (LYVE-1 and PROX1) of epicardial lymphatic vessels in the model of dilated cardiomyopathy induced by Adriamycin were significantly lower than those in normal rats according to the immunohistochemistry results, and it could be effectively reversed by KXF, which also indicates the presence of impaired lymphatic vessels in dilated cardiomyopathy. According to the results of our study, regulating lymphangiogenesis would be a breakthrough in treating dilated cardiomyopathy, and Kuoxin Decoction is an effective formula in traditional Chinese medicine.

## Limitation

KXF has been shown to stimulate the mRNA expression of *VEGF-A/C*, but we could not observe the protein expression of lymphangiogenesis in zebrafish because of lacking antibodies suitable for western blot assay in the zebrafish model in the market. We only studied the expressions of the protein levels of VEGF-C and VEGFR-3 in lymphatic endothelial cells instead of zebrafish. The further pharmacological action and mechanism of KXF in promoting lymphangiogenesis will be studied in the future.

## Conclusion

Network analysis results showed that KXF could regulate lymphangiogenesis through the VEGF family. The prediction that KXF could promote lymphangiogenesis has been verified in the zebrafish model and lymphatic endothelial cells. The mechanism may be related to the upregulation of VEGF-C/VEGFR-3 expression, and the primary component that exerts this effect may be salvianolic acid B.

## Data Availability

The original contributions presented in the study are included in the article/Supplementary Material; further inquiries can be directed to the corresponding authors.

## Abbreviations

BP, biological process; CC, cellular component; CCK-8, Cell Counting Kit-8; DCM, dilated cardiomyopathy; DL, drug-likeness; GO, Gene Ontology; KEGG, Kyoto Encyclopedia of Genes and Genomes; KXF, Kuoxin Decoction; LECs, lymphatic endothelial cells; MF, molecular function; OB, human oral bioavailability; PPI, protein–protein interaction networks; SAB, salvianolic acid B; VEGFR-3, vascular endothelial growth factor receptor-3.
