# Supplementary figures and images for: Kuoxin Decoction promotes lymphangiogenesis in zebrafish and in vitro based on network analysis
Source: Front Pharmacol. 2022 Aug 11;13:915161. doi: 10.3389/fphar.2022.915161 (PMC9465995; doi:10.3389/fphar.2022.915161)

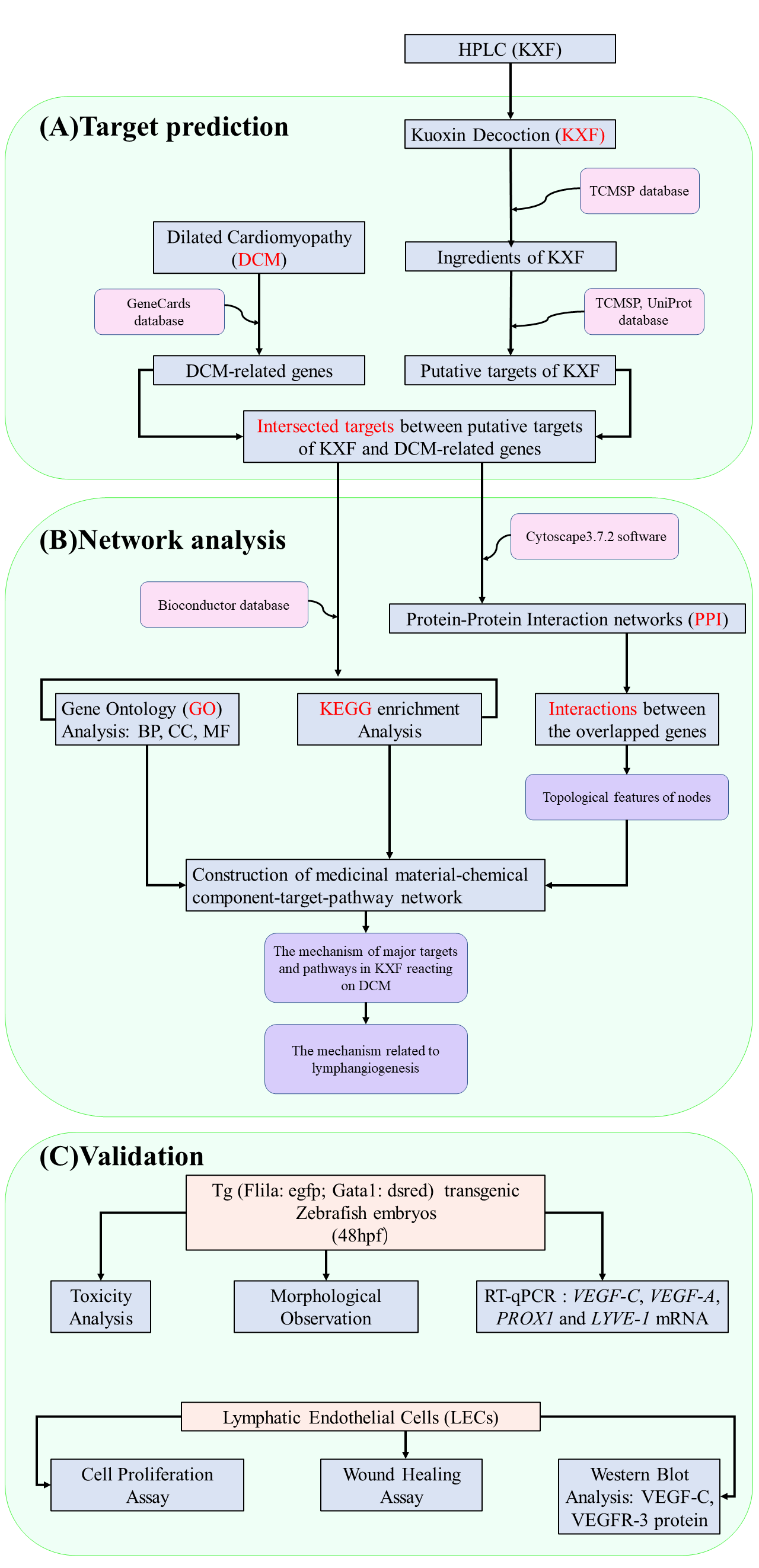

Supplement: Supplementary file 2 [file Image1.TIF]
